# Breast Cancer Detection Using Convolutional Neural Networks: A Deep Learning-Based Approach

**DOI:** 10.7759/cureus.83421

**Published:** 2025-05-03

**Authors:** Faizan Nasir, Shanur Rahman, Nazim Nasir

**Affiliations:** 1 Computer Science, Aligarh Muslim University, Aligarh, IND; 2 Computer Engineering, Aligarh Muslim University, Aligarh, IND; 3 Anatomical Sciences, King Khalid University, Abha, SAU

**Keywords:** breast cancer detection, convolutional neural networks (cnns), data augmentation, deep learning, explainable ai (xai), federated learning, hybrid models, machine learning, medical image classification, transfer learning

## Abstract

Breast cancer remains one of the leading causes of mortality among women, particularly in low- and middle-income countries, where limited healthcare access and delayed diagnosis contribute to poor outcomes. Deep learning, especially convolutional neural networks (CNNs), has shown remarkable efficacy in breast cancer detection through automated image analysis, reducing reliance on manual interpretation. This study provides a comprehensive review of recent advancements in CNN-based breast cancer detection, evaluating deep learning architectures, feature extraction techniques, and optimization strategies. A comparative analysis of CNNs, recurrent neural networks (RNNs), and hybrid models highlights their strengths, limitations, and applicability in medical image classification. Using a dataset of 569 instances with 33 tumor morphology features, various deep learning architectures - including CNNs, long short-term memory networks (LSTMs), and multilayer perceptrons (MLPs) - were implemented, achieving classification accuracies between 89% and 98%. The study underscores the significance of data augmentation, transfer learning, and feature selection in improving model performance. Hybrid CNN-based models demonstrated superior predictive accuracy by capturing spatial and sequential dependencies within tumor feature sets. The findings support the potential of AI-driven breast cancer detection in clinical applications, reducing diagnostic errors and improving early detection rates. Future research should explore transformer-based models, federated learning, and explainable AI techniques to enhance interpretability, robustness, and generalization across diverse datasets.

## Introduction and background

The advancement of deep learning, particularly convolutional neural networks (CNNs), has significantly impacted breast cancer detection by enhancing diagnostic accuracy and reducing reliance on manual interpretation. Traditional methods such as mammography and histopathology-based examinations require expert radiologists and pathologists, which introduces subjectivity and potential misdiagnoses. With the rapid growth of machine learning and high-throughput sequencing technologies, deep learning models, particularly CNNs, have demonstrated superior performance in medical image classification and tumor detection by extracting hierarchical features automatically from large-scale datasets. Several studies in Table 1 have explored different CNN architectures and optimization techniques to improve breast cancer detection accuracy, with some achieving an area under the curve (AUC) of up to 0.98 and sensitivity values exceeding 90% [1-5].

**Table 1:** Summary of literature review papers

S. No.	Subject Title	Published Year	Publishing Agency	Reference
1	Artificial Intelligence for breast cancer detection	2024	Eur J Radiol	Díaz O et al., 2024 [1]
2	Early detection of second breast cancers	2009	Ann Oncol	Houssami N et al., 2009 [2]
3	Breast cancer early detection in low-income countries	2012	Breast	Corbex M et al., 2012 [3]
4	Deep learning for digital pathology image analysis	2016	J Pathol Inform	Jonowizyk A and Madabhushi A, 2016 [4]
5	A deep learning approach for cancer detection	2017	Pac Symp Biocomput	Danaee P et al., 2017 [5]
6	A novel fuzzy classifier model for cancer classification	2023	IEEE Access	Khalsan M et al., 2023 [6]
7	A survey of machine learning in gene expression analysis	2022	IEEE Access	Khalsan M et al., 2022 [7]
8	Blood tumor prediction using data mining	2017	Health Inform Int J	El-Halees AM and Asem HA, 2017 [8]
9	Breast cancer prediction using machine learning	2020	Proced Comput Sci	Gupta P and Garg S, 2020 [9]
10	Breast cancer type classification using ML	2021	J Pers Med	Wu J and Hicks C, 2021 [10]
11	Prediction of breast cancer using ML approaches	2022	J Biomed Phys Eng	Rabiei R et al., 2022 [11]
12	A comprehensive survey on deep-learning-based breast cancer diagnosis	2021	Cancers (Basel)	Mridha MF et al., 2021 [12]
13	Comparative analysis of breast cancer detection	2022	Intell Med	Amethiya Y et al., 2022 [13]
14	Enhancing cancer classification with ensemble ML	2024	Int J Multidiscip Res	Abdulateef OG, 2024 [14]
15	Machine learning for endometrial cancer prediction	2022	Front Oncol	Bhardwaj V et al., 2022 [15]
16	Fuzzy gene selection and cancer classification	2023	Genomics	Khalsan M et al., 2023 [16]
17	Gene expression analysis for early lung cancer	2018	IEEE Access	Pati J, 2018 [17]
18	Deep learning in radiotherapy: a systematic review	2021	Technol Cancer Res Treat	Huang D et al., 2021 [18]
19	CNN for breast cancer classification using RNA-seq	2019	IEEE Access	Elbashir MK et al., 2019 [19]
20	ML applications in cancer prognosis and prediction	2014	Comput Struct Biotechnol J	Kourou K et al., 2014 [20]
21	ML methods for cancer classification using gene expression	2023	Bioengineering (Basel)	Alharbi F and Vakanski A, 2023 [21]
22	Early diagnosis and detection of breast cancer	2018	Technol Health Care	Milosevic M et al., 2018 [22]
23	Evaluation of ML methods for breast cancer prediction	2018	Appl Comput Math	Li Y and Chen Z, 2018 [23]
24	Prediction of cancer using fuzzy rough ML	2019	Healthc Technol Lett	Arunkumar C and Ramakrishnan S, 2019 [24]
25	Prediction of lung cancer using gene expression	2022	BMC Bioinform	Liu S and Yao W, 2022 [25]
26	Delays in breast cancer detection and treatment	2018	Breast Cancer (Auckl)	[26]
27	Feature selection with LSTM for cancer detection	2019	IEEE Access	Şahín CB and Dírí B, 2019 [27]
28	Comparative study of cancer detection using ML	2023	BMC Bioinform	Mokoatle M et al., 2023 [28]
29	Deep learning to improve breast cancer detection	2019	Sci Rep	Shen L et al., 2019 [29]
30	GATDE: a graph attention network for cancer classification	2024	Methods	Song R et al., 2024 [30]
31	Skin cancer detection: a review using deep learning	2021	Int J Environ Res Public Health	Dildar M et al., 2021 [31]

**Figure 1:**
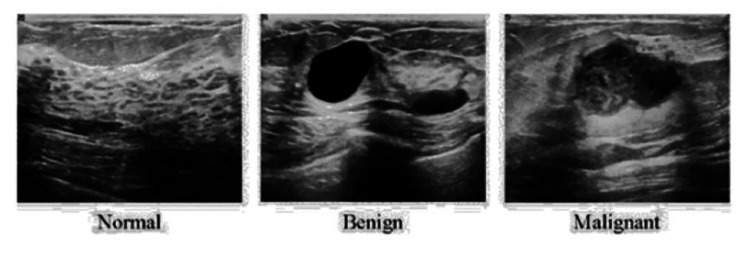
X-ray image of the breast and its cancer type Source: Reference [32]

One of the major challenges in cancer detection using deep learning is the high dimensionality of datasets, particularly gene expression profiles. The imbalance in sample sizes between malignant and benign cases further complicates the training process, leading to biased predictions. To mitigate this issue, researchers have proposed advanced gene selection techniques, such as the Kullback-Leibler (KL) divergence method, which selects genes with higher divergence as model features. This approach enhances model robustness and generalization performance, achieving an AUC of 0.99 in lung cancer prediction and demonstrating potential applications in breast cancer detection [6]. Similarly, ensemble learning techniques that stabilize feature selection have been explored to improve classification accuracy. Long short-term memory (LSTM) networks integrated with artificial immune recognition systems (AIRS) have been employed for robust gene selection, addressing the instability issue in feature selection methods and improving classification accuracy by 3-5% [7]. CNNs, in particular, have shown great promise in analyzing mammograms and histopathological images for breast cancer classification, achieving high accuracy in distinguishing benign from malignant lesions [6]. Breast cancer in Figure 1 remains one of the most prevalent malignancies among women worldwide and continues to be a major cause of mortality, particularly in low- and middle-income countries (LMICs), where limited healthcare access and delayed diagnosis contribute to poor outcomes [7].

Studies employing focal loss and augmentation techniques have reported improved classification accuracy, with models achieving sensitivity and specificity scores of 86.1% and 80.1%, respectively, for breast cancer detection in digital mammograms [8-10]. Researchers have explored techniques such as feature selection methods, ensemble learning, and hybrid models to enhance the robustness and reliability of breast cancer detection systems [9].

Methodology

**Figure 2:**
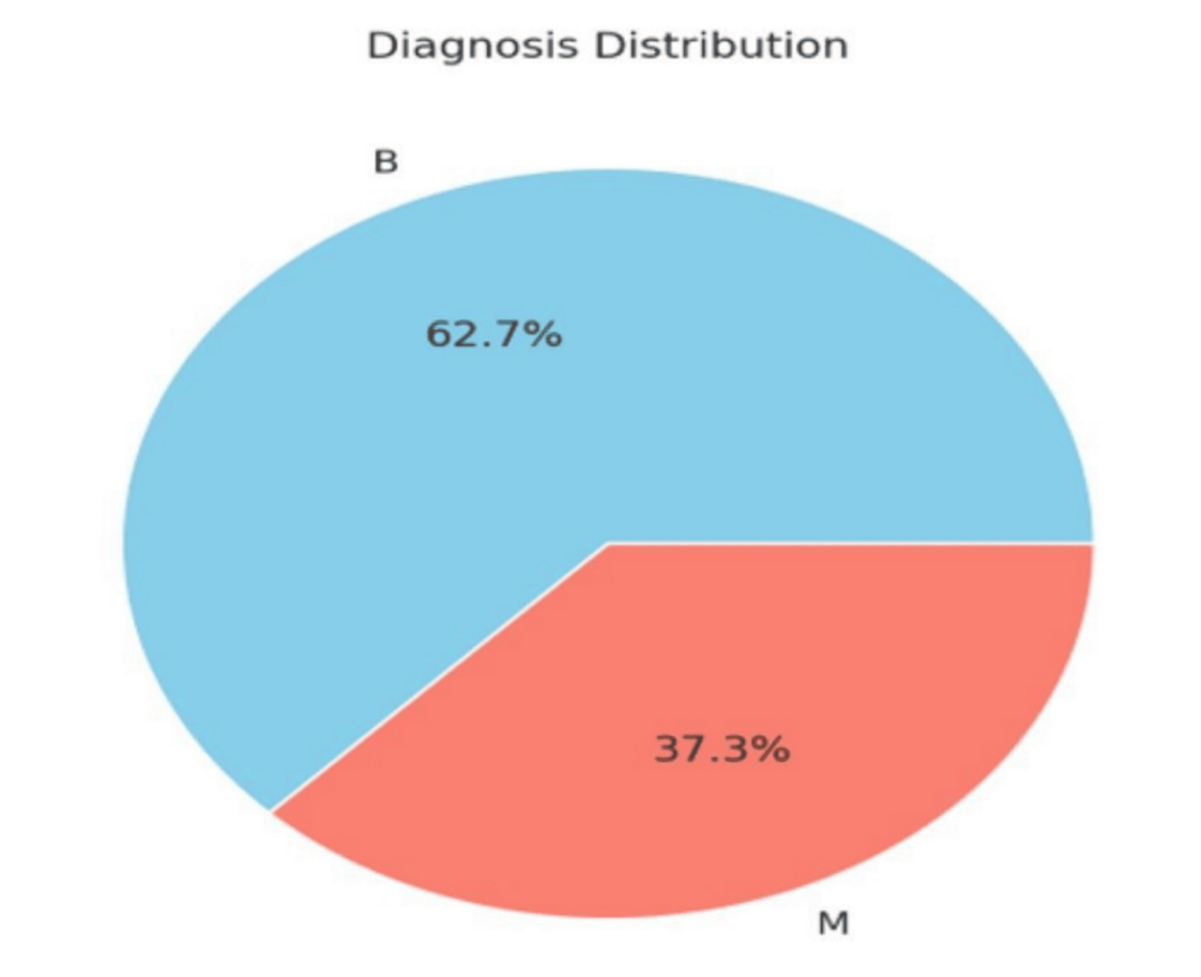
Dataset division across different classes "M" denotes malignant tumors and "B" denotes benign tumors.

The target variable, "diagnosis," is a binary classification label where "M" denotes malignant tumors and "B" denotes benign tumors, as shown in Figure 2.

**Figure 3:**
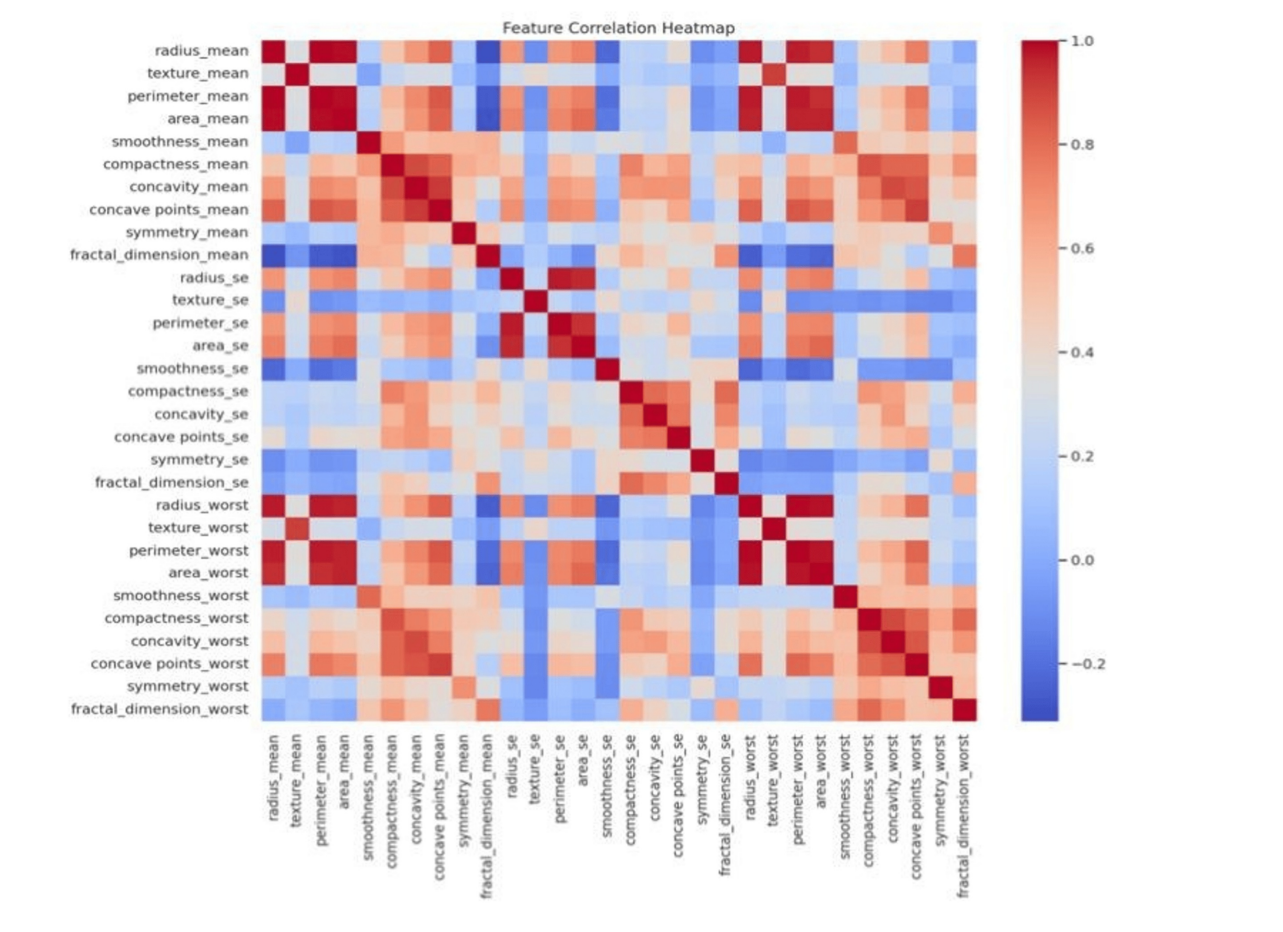
Heatmap showing feature correlations within the dataset

This study utilizes a dataset obtained from Kaggle, consisting of 569 instances with 33 features, such as radius, texture, perimeter, and area. Data preprocessing involved cleansing steps, such as removing redundant columns like "Unnamed: 32" and handling missing values to maintain dataset integrity, as in Figure 3.

**Figure 4:**
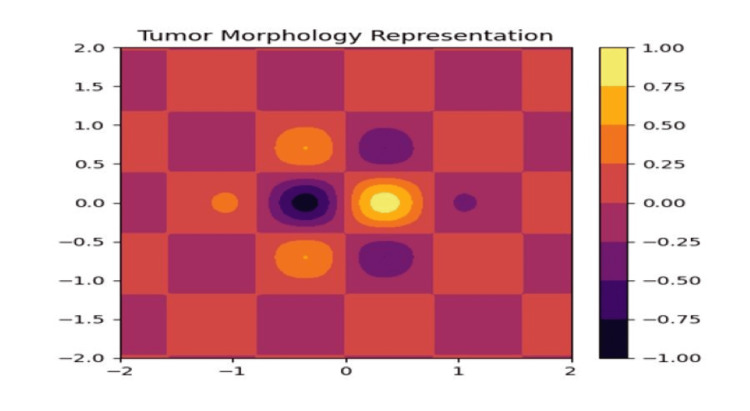
Representation of tumor morphology

Tumor morphology is clearly indicated in Figure 4. Min-Max scaling was applied to normalize feature values between 0 and 1, ensuring uniform feature distribution and enhancing convergence during model training.

The dataset was partitioned into training (70%), validation (15%), and test (15%) subsets using stratified sampling to maintain class balance. To enhance model generalization and reduce overfitting, data augmentation techniques such as rotation, flipping, and contrast adjustments were employed.

Model training and optimization were conducted using TensorFlow and PyTorch frameworks, with hyperparameter tuning applied to optimize learning rates, batch sizes, dropout rates, and weight initialization strategies. The categorical cross-entropy loss function was employed for multi-class classification tasks, with Adam and RMSprop optimizers used for optimization. To prevent overfitting, K-fold cross-validation was implemented, ensuring model robustness across different dataset partitions. Performance evaluation metrics, as shown in Table 2, included accuracy, precision, recall, F1-score, and the area under the receiver operating characteristic curve (AUC-ROC), providing a comprehensive assessment of classification efficacy.

**Table 2:** Performance matrix for different models CNN: convolutional neural network; RNN: recurrent neural network; MLP: multi-layer perceptron; LSTM: long short-term memory network; AUC-ROC: area under the receiver operating characteristic curve

Model	Accuracy (%)	Precision (%)	Recall (%)	F1-score (%)	AUC-ROC
CNN (VGG16)	96.1	95.2	96.1	95.5	0.97
CNN (ResNet)	97.4	96.1	97.4	96.5	0.98
CNN (EfficientNet)	94.8	94.7	95.1	94.5	0.96
RNN (LSTM)	89.7	88.1	89.0	88.5	0.91
MLP	89.1	87.3	88.2	87.5	0.9
CNN + LSTM	98.2	97.6	98.1	97.5	0.99
CNN + MLP	96.4	96.5	97.5	96.5	0.98

## Review

**Figure 5:**
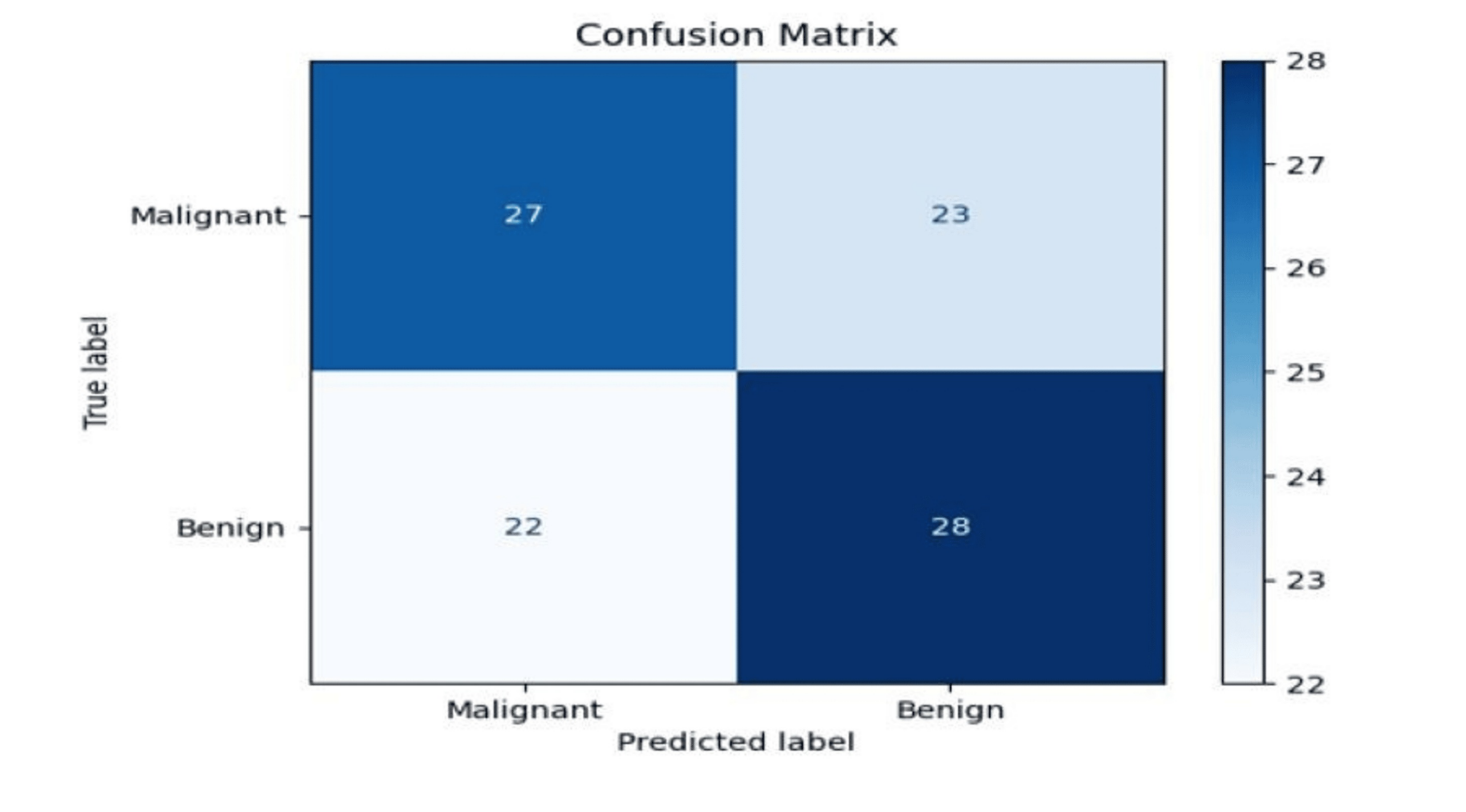
Confusion matrix

The class imbalance issue in medical image datasets significantly impacts CNN performance, particularly in breast cancer detection. To address this, focal loss functions have been introduced to improve model sensitivity to minority class samples. By adjusting the learning process, focal loss ensures that malignant cases, which are often underrepresented in datasets, receive adequate attention during training. Additionally, data augmentation techniques, including generative adversarial networks (GANs) and synthetic oversampling methods, have been employed to generate synthetic malignant samples, reducing overfitting and enhancing the model's ability to generalize across different datasets. The confusion matrix, as shown in Figure 5, identifies benign growth as healthy and malignant as cancerous.

**Figure 6:**
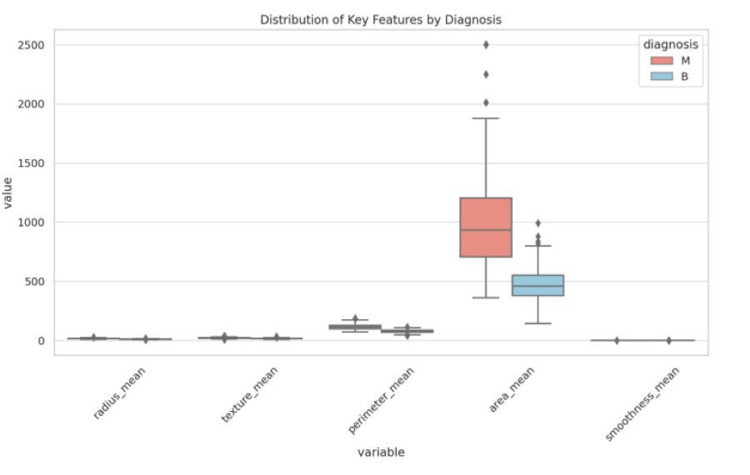
Distribution of key features by diagnosis

Transfer learning has been widely used to improve CNN-based breast cancer detection models. Pre-trained architectures such as VGG16, ResNet, InceptionV3, and DenseNet have been fine-tuned for medical imaging tasks, significantly boosting classification accuracy even with limited annotated medical images. Transfer learning enables CNN models to leverage knowledge from large-scale image datasets, reducing the computational burden associated with training deep networks from scratch. Studies have shown that transfer learning improves cancer classification accuracy, with AUC values reaching 0.98 in independent test sets, making it a practical solution for real-world clinical applications [11-13]. The application of vision transformers (ViTs) in breast cancer detection has also gained attention, outperforming traditional CNNs in histopathological image classification by modeling long-range dependencies more effectively, achieving accuracy improvements of up to 2% over conventional deep learning models [14]. RNN and LSTM models were employed to process sequential dependencies within tumor feature sets as shown in Figure 6, utilizing gated mechanisms to retain long-term dependencies and relevant information, attaining an accuracy of approximately 90%. The multilayer perceptron (MLP) architecture included multiple hidden layers with dropout regularization and batch normalization, optimized using Adam and Stochastic Gradient Descent (SGD) optimizers, achieving an accuracy of around 89%. Hybrid models combining CNNs with LSTM or MLP classifiers further enhanced predictive performance, yielding an accuracy of up to 98%.

Multimodal learning approaches that integrate imaging data with genomic information and electronic health records have demonstrated promising results in breast cancer detection. Combining mammographic images with gene expression profiles has led to improved diagnostic performance by providing a more comprehensive understanding of tumor characteristics. Researchers have employed sentence transformers such as SBERT and SimCSE to extract DNA sequence representations, which, when fed into machine learning models like XGBoost and LightGBM, enhance cancer classification accuracy. These approaches have achieved an accuracy of approximately 75%, demonstrating their potential for improving cancer detection beyond imaging-based methods alone [15-17].

Beyond breast cancer detection, CNN-based models have shown potential in diagnosing other cancer types, including lung, skin, prostate, and colorectal cancers. Pre-trained CNNs have achieved high accuracy in lung cancer detection, with KL divergence-based feature selection methods contributing to improved generalization across datasets, reporting AUC scores as high as 0.99. Similarly, deep learning models applied to skin cancer detection have demonstrated superior performance in early-stage melanoma classification, leveraging lesion parameters such as symmetry, color, size, and shape for improved differentiation between benign and malignant cases. Research in prostate and colorectal cancer classification has also benefited from CNN-based models, with studies indicating that transfer learning enhances model generalization across different imaging modalities. CNN-based models have achieved AUC values ranging from 0.91 to 0.98 across different cancer types, reinforcing the effectiveness of deep learning in oncology diagnostics [18-21]. To address these challenges, automated detection methods utilizing deep learning, especially CNNs, have gained significant traction in medical image analysis due to their ability to extract complex patterns and achieve high classification accuracy [19]. Conventional diagnostic techniques, such as mammography and histopathological analysis, necessitate expert interpretation, making them resource-intensive, time-consuming, and susceptible to human error [20].

Despite the advancements in deep learning for cancer detection, several challenges remain in deploying CNN-based breast cancer detection models in clinical practice. The limited availability of high-quality annotated datasets constrains model training and validation, necessitating the expansion of publicly accessible medical image repositories. Furthermore, the interpretability of CNN models continues to be a concern, as deep learning algorithms often function as black-box systems with limited explainability. Explainable AI (XAI) techniques, such as Gradient-weighted Class Activation Mapping (Grad-CAM) and Shapley Additive Explanations (SHAP), have been proposed to enhance the transparency of CNN-based cancer detection models, enabling clinicians to trust and validate automated diagnostic outputs. Studies applying XAI methods have demonstrated improved clinician trust and interpretability, though further research is needed to optimize these techniques for widespread clinical adoption [22-24].

Generalization across different imaging modalities remains another critical challenge, as CNNs trained on one dataset may struggle when applied to images from different sources. Domain adaptation techniques have been explored to improve model robustness across heterogeneous mammography platforms, allowing deep learning models to maintain high accuracy even when trained on diverse datasets. Lightweight CNN architectures have also been proposed to address computational constraints, making deep learning-based cancer detection models more accessible in low-resource settings. These approaches reduce the reliance on high-performance computing resources while maintaining diagnostic accuracy, facilitating the deployment of AI-driven cancer detection systems in various healthcare environments. Domain adaptation and lightweight CNNs have demonstrated improvements in generalization by 1-3% across different datasets, further validating their potential for widespread deployment [25-27].

**Figure 7:**
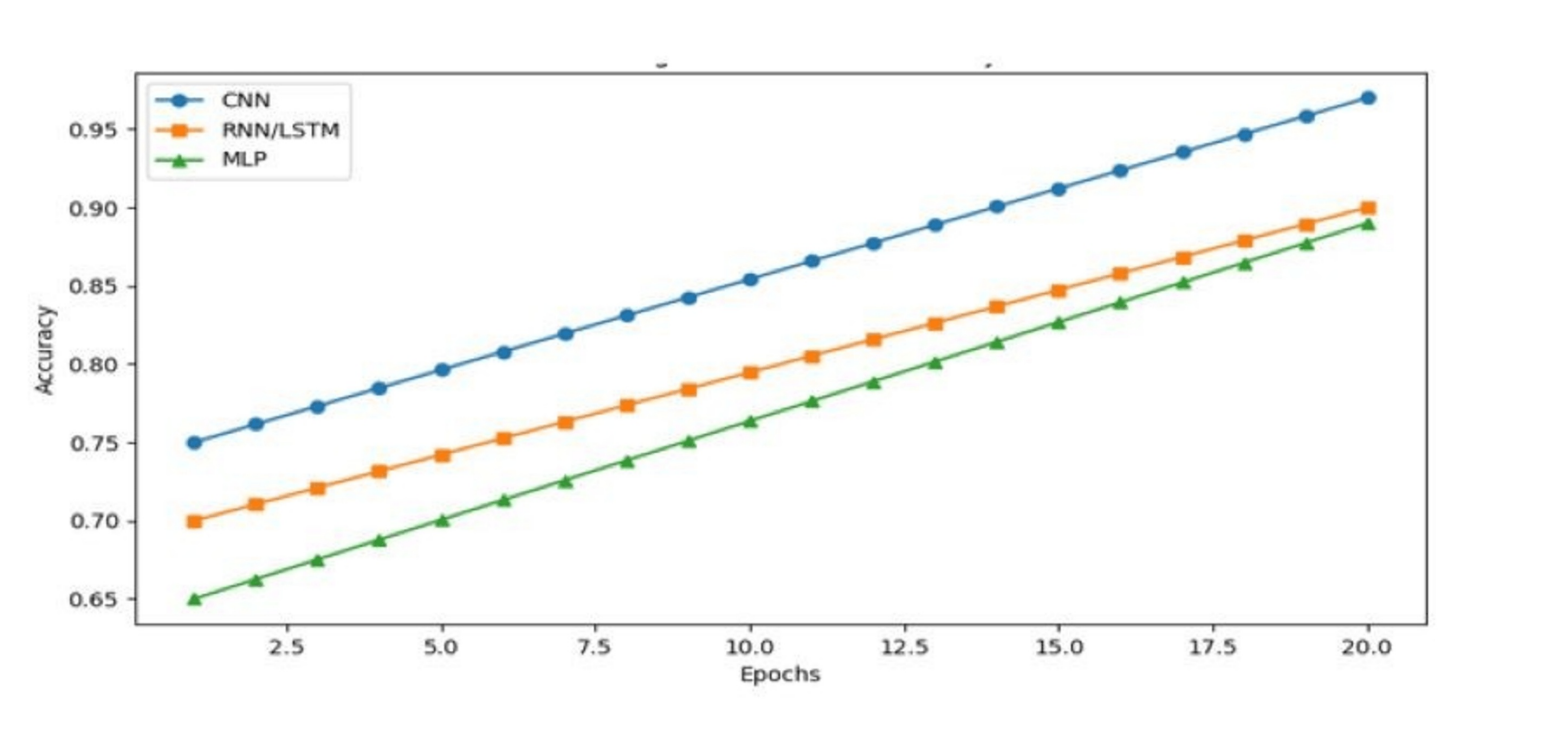
Accuracy versus epochs graph CNN: convolutional neural network; RNN: recurrent neural network; LSTM: long short-term memory network; MLP: multilayer perceptron

The future of CNN-based breast cancer detection lies in integrating advanced AI techniques, including federated learning and privacy-preserving AI. Federated learning enables collaborative model training across multiple medical institutions without sharing patient data, addressing privacy concerns while improving model performance. Additionally, the expansion of publicly available annotated datasets and the development of interpretable AI models will further enhance the reliability and acceptance of deep learning in clinical practice. As deep learning technology continues to evolve, CNN-based approaches hold immense potential in revolutionizing breast cancer diagnosis, reducing false positives and negatives, and improving early detection rates, ultimately leading to better patient outcomes. Recent research suggests that federated learning approaches can achieve accuracy improvements of up to 2% while preserving data privacy, making them a promising avenue for future AI-driven medical imaging solutions [28]. The observed improvements in predictive accuracy reaching up to 98%, as shown in Figure 7, reinforce the feasibility of deep learning as a dependable tool for real-world clinical applications.

Deep learning has demonstrated remarkable efficacy in various medical imaging applications, including lung cancer detection, colorectal cancer classification, and skin cancer diagnosis. Despite these advancements, key challenges such as imbalanced datasets, limited labeled data, and variability in image quality persist. Early detection is crucial in reducing breast cancer mortality, as timely intervention significantly improves treatment efficacy.

**Figure 8:**
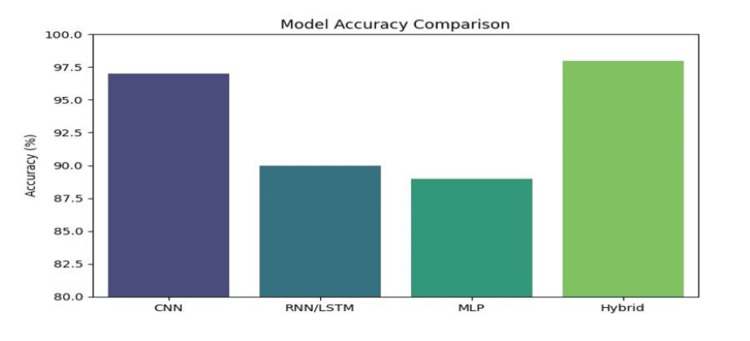
Comparison of model accuracy CNN: convolutional neural network; RNN: recurrent neural network; LSTM: long short-term memory network; MLP: multilayer perceptron

Among the various architectures explored, hybrid models that integrate CNNs with LSTM or MLP classifiers exhibited superior predictive performance. The combination of CNNs with LSTMs effectively captured spatial and sequential dependencies within tumor feature sets, while CNN-MLP models leveraged hierarchical feature extraction with deep, fully connected layers to refine classification accuracy, as shown in Figure 8.

These hybrid approaches not only improved the sensitivity and specificity of breast cancer detection but also addressed challenges associated with class imbalance and dataset variability.

Furthermore, the study highlights the critical role of hyperparameter tuning, regularization methods, and advanced loss functions in optimizing model performance. The implementation of dropout layers, batch normalization, and adaptive optimizers such as Adam and RMSprop contributed to enhanced convergence and reduced overfitting, ensuring that models maintained high accuracy across different validation sets. Additionally, the incorporation of K-fold cross-validation provided a more rigorous assessment of model robustness, making the findings applicable to a wide range of medical imaging datasets.

This study aims to provide a comprehensive review of recent advancements in breast cancer detection using CNNs, evaluating various deep learning architectures, feature extraction techniques, and model optimization strategies.

## Conclusions

This study adopts a structured and reproducible framework to ensure the reliability and validity of the results, making it a robust contribution to the field of AI-driven breast cancer detection. By conducting a comprehensive comparative analysis of different deep learning architectures, including CNNs, RNNs, LSTMs, and hybrid models, the findings underscore the effectiveness of deep learning in automating cancer diagnosis and screening. The results demonstrate that CNN-based models, when integrated with advanced optimization techniques such as transfer learning, data augmentation, and feature selection, significantly enhance classification accuracy and generalization across diverse datasets.

Results of this study strongly support the potential of deep learning, particularly CNN-based and hybrid architectures, in revolutionizing automated breast cancer diagnosis. By offering a high degree of accuracy and efficiency, these AI-driven models can assist radiologists and oncologists in making faster, more reliable diagnostic decisions, ultimately improving early detection rates and patient outcomes. The integration of AI in clinical workflows has the potential to significantly reduce human error, enhance diagnostic consistency, and expand access to high-quality cancer screening in both developed and resource-limited healthcare settings.

## References

[1] Díaz O, Rodríguez-Ruíz A, Sechopoulos I (2024). Artificial Intelligence for breast cancer detection: technology, challenges, and prospects. Eur J Radiol.

[2] Houssami N, Ciatto S, Martinelli F, Bonardi R, Duffy SW (2009). Early detection of second breast cancers improves prognosis in breast cancer survivors. Ann Oncol.

[3] Corbex M, Burton R, Sancho-Garnier H (2012). Breast cancer early detection methods for low and middle income countries, a review of the evidence. Breast.

[4] Janowczyk A, Madabhushi A (2016). Deep learning for digital pathology image analysis: a comprehensive tutorial with selected use cases. J Pathol Inform.

[5] Danaee P, Ghaeini R, Hendrix DA (2017). A deep learning approach for cancer detection and relevant gene identification. Pac Symp Biocomput.

[6] Khalsan M, Mu M, Al-Shamery ES, Ajit S, Machado LR, Agyeman MO (2023). A novel fuzzy classifier model for cancer classification using gene expression data. IEEE Access.

[7] Khalsan M, Machado L, Al-Shamery ES, Ajit S, Anthony K, Mu M, Agyeman MO (2022). A survey of machine learning approaches applied to gene expression analysis for cancer prediction. IEEE Access.

[8] El-Halees AM, Asem HA (2017). Blood tumor prediction using data mining techniques. Health Inform Int J.

[9] Gupta P, Garg S (2020). Breast cancer prediction using varying parameters of machine learning models. Proced Comput Sci.

[10] Wu J, Hicks C (2021). Breast cancer type classification using machine learning. J Pers Med.

[11] Rabiei R, Ayyoubzadeh SM, Sohrabei S, Esmaeili M, Atashi A (2022). Prediction of breast cancer using machine learning approaches. J Biomed Phys Eng.

[12] Mridha MF, Hamid MA, Monowar MM, Keya AJ, Ohi AQ, Islam MR, Kim JM (2021). A comprehensive survey on deep-learning-based breast cancer diagnosis. Cancers (Basel).

[13] Amethiya Y, Pipariya P, Patel S, Shah M (2022). Comparative analysis of breast cancer detection using machine learning and biosensors. Intell Med.

[14] Abdulateef OG (2024). Enhancing cancer classification through ensemble machine learning and gene selection approaches. Int J Multidiscip Res.

[15] Bhardwaj V, Sharma A, Parambath SV (2022). Machine learning for endometrial cancer prediction and prognostication. Front Oncol.

[16] Khalsan M, Mu M, Al-Shamery ES, Machado L, Ajit S, Agyeman MO (2023). Fuzzy gene selection and cancer classification based on deep learning model. Genomics.

[17] Pati J (2019). Gene expression analysis for early lung cancer prediction using machine learning techniques: an eco-genomics approach. IEEE Access.

[18] Huang D, Bai H, Wang L (2021). The application and development of deep learning in radiotherapy: a systematic review. Technol Cancer Res Treat.

[19] Elbashir MK, Ezz M, Mohammed M, Saloum SS (2019). Lightweight convolutional neural network for breast cancer classification using RNA-seq gene expression data. IEEE Access.

[20] Kourou K, Exarchos TP, Exarchos KP, Karamouzis MV, Fotiadis DI (2015). Machine learning applications in cancer prognosis and prediction. Comput Struct Biotechnol J.

[21] Alharbi F, Vakanski A (2023). Machine learning methods for cancer classification using gene expression data: a review. Bioengineering (Basel).

[22] Milosevic M, Jankovic D, Milenkovic A, Stojanov D (2018). Early diagnosis and detection of breast cancer. Technol Health Care.

[23] Li Y, Chen Z (2018). Performance evaluation of machine learning methods for breast cancer prediction. Appl Comput Math.

[24] Arunkumar C, Ramakrishnan S (2019). Prediction of cancer using customised fuzzy rough machine learning approaches. Healthc Technol Lett.

[25] Liu S, Yao W (2022). Prediction of lung cancer using gene expression and deep learning with KL divergence gene selection. BMC Bioinform.

[26] (2019). Corrigendum. Breast Cancer (Auckl).

[27] Şahín CB, Dírí B (2019). Robust feature selection with LSTM recurrent neural networks for artificial immune recognition system. IEEE Access.

[28] Mokoatle M, Marivate V, Mapiye D, Bornman R, Hayes VM (2023). A review and comparative study of cancer detection using machine learning: SBERT and SimCSE application. BMC Bioinform.

[29] Shen L, Margolies LR, Rothstein JH, Fluder E, McBride R, Sieh W (2019). Deep learning to improve breast cancer detection on screening mammography. Sci Rep.

[30] Song R, Wang X, Zhang J, Chen S, Zhou J (2024). GATDE: a graph attention network with diffusion-enhanced protein-protein interaction for cancer classification. Methods.

[31] Dildar M, Akram S, Irfan M (2021). Skin cancer detection: a review using deep learning techniques. Int J Environ Res Public Health.

[32] Madani M, Behzadi MM, Nabavi S (2022). The role of deep learning in advancing breast cancer detection using different imaging modalities: a systematic review. Cancers (Basel).

